# Cold-Pressed Okra Seed Oil Byproduct as an Ingredient for Muffins to Decrease Glycemic Index, Maillard Reaction, and Oxidation

**DOI:** 10.1021/acsomega.3c06027

**Published:** 2024-02-09

**Authors:** Alican Akcicek, Muhammed Özgölet, Zeynep Hazal Tekin-Cakmak, Salih Karasu, Esra Duran, Osman Sagdic

**Affiliations:** † Faculty of Tourism Department of Gastronomy and Culinary Arts, Kocaeli University, Kartepe, Kocaeli 41080, Turkey; ‡ Faculty of Chemical and Metallurgical Engineering, Department of Food Engineering, Yildiz Technical University, 34225 Istanbul, Turkey; § Department of Nutrition and Dietetics, Istanbul Arel University, Faculty of Health Sciences, 34537 Istanbul, Turkey

## Abstract

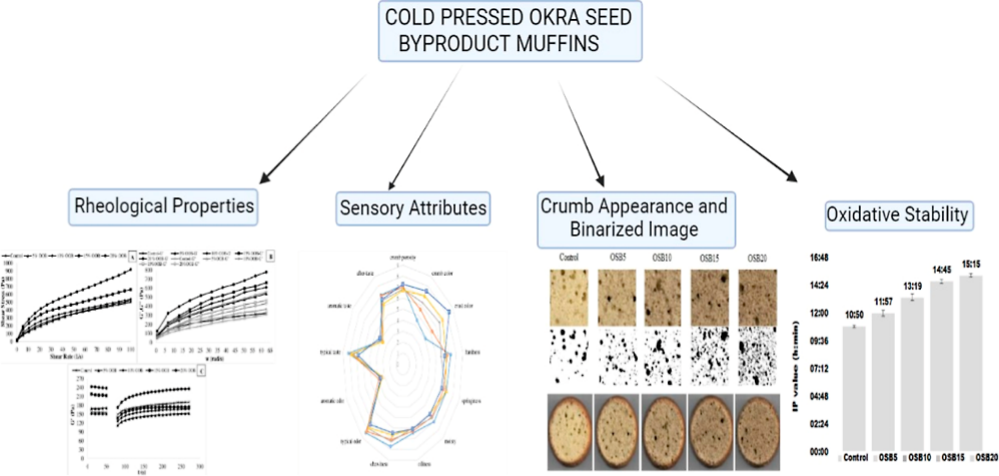

This study aimed to investigate the effects of adding cold-pressed okra seed oil byproduct (OSB) to the muffin formulation, as a partial substitute for wheat flour, on the nutritional, physicochemical, rheological, textural, and sensory properties of muffins. The carbohydrate, protein, oil, moisture, and ash contents of OSB were 44.96, 32.34, 10.21, 7.51, and, 4.98%, respectively, indicating that OSB was rich in protein and carbohydrate. All muffin samples showed a shear thinning behavior, indicating that the viscosity of all samples decreased with increasing shear rate. The frequency sweep test showed that all samples showed viscoelastic solid-like structure [*G*′ (storage modulus)> *G*″ (loss modulus)]. The *K*′ values (between 66.45 and 139.14) were higher than the *K*″ values (between 36.62 and 80.42) for all samples. The result was another indication of the viscoelastic solid characteristic of the samples. In our study, it was found that the fluorescence of advanced Maillard products and soluble tryptophan index decreased with increasing amount of OSB, indicating that OSB addition led to a decrease in the amount of fluorescent Maillard reaction (MR) products. The fortified muffins with more than 10% OSB had a reduced estimated glycemic index (GI) significantly in comparison with control muffin samples (*p* < 0.05). The induction period (IP) values of the muffin samples containing OSB (between 11:57 and 15:15 h/min) were higher than the IP value of the control sample (10:50 h/min), indicating that OSB improved the oxidative stability of the muffin samples. The addition of OSB has shown no negative effect on sensory attributes considering texture, mouth fell, odor, and taste. This study suggested that the addition of OSB in muffins could improve rheological properties and oxidative stability and decrease GI and the amount of MR products without negative impact on sensory properties.

## 1 Introduction

Growing regions for okra seed (Hibiscus esculentus) include Africa, Asia, Southern Europe, the Mediterranean region, and North America. Oil and protein were obtained from okra seeds. On a modest scale, oil has been produced from okra seeds. András, Simándi, Örsi, Lambrou, Missopolinou-Tatala, Panayiotou, Domokos, and Doleschall^1^ reported the fresh okra seed from Greece to be a potential source of oil with concentrations varying from 15.9 to 20.7%. The oil mainly consisted of linoleic acid (up to 47.4%).^1^ Okra seeds, which represent about 13.5% in dried okra,^2^ are a rich source of high-quality proteins and oil, mainly consisting of linoleic acid.^3^ Savhlo, Martins and Hull^4^ found okra seed oil as one of the rich sources of unsaturated fatty acids. Karakoltsidis and Constantinides^5^ reported that okra seed was investigated for the first time for its potential as a seed protein. The protein content of okra seed is as high as 45% after extraction of oil.^6^ Also, okra seeds are rich in phenolic compounds and were mainly composed of oligomeric catechins (2.5 mg/g of seeds) and flavonol derivatives (3.4 mg/g of seeds).^2^ The seeds are cultivated throughout the tropical and warm regions, and it is among the most heat- and drought-tolerant vegetable species in the world. The immature fibrous fruits containing round, white seeds are mainly consumed as a fresh (or quick-frozen) vegetable and are a good source of nutrients and bioactive compounds, such as dietary fiber and phenolics.^7^

Cold pressing is a technique that does not require any complex process with cheap and high-quality oil production and is performed by applying only a conventional screw press for the continuation of the pressing process. Since there is no solvent usage during pressing, no heat treatment is applied, and the oils are not subjected to any of the refining process steps, it is stated that while all of the bioactive components are naturally contained in the oils produced by this method, they do not contain any residue.^8,9^ Therefore, the physicochemical and organoleptic properties and bioactive components (e.g., flavonoids and phenolic acids) of the cold-pressed oils are preserved. Therefore, cold-pressed oils have drawn more and more attention from customers.^10,11^

Cold press extraction of seed oils, which have a very rich nutritional content, is especially preferred for the food, pharmaceutical, and cosmetic industries, and this has led to obtain high amount of valuable byproducts.^12^ Karaman, Karasu, Tornuk, Toker, Gecgel, Sagdic, Ozcan, and Gul^13^ investigated the functionality and nutritional properties of cold-pressed oil byproducts. Cold-pressed byproducts were used in salad dressing, ice cream, vegan mayonnaise samples, and muffin for a nutritional property, fat replacer, and stabilizer.^14−18^ Because of its high nutritional quality, cold-pressed okra seed oil byproduct (OSB) has not yet been used in any food formulation, so it can be a potential ingredient in muffin formulation, which is highly preferred by consumers.

While muffins and other sweet baked goods generally contain high amounts of protein, fat, and carbohydrate, which are avoided by health-conscious consumers, they have lower contents of vitamins, minerals, phenolic compounds, and dietary fiber. Therefore, studies on increasing the nutritional value of muffins have been increased.^19,20^ With the addition of dietary fiber, the functional feature of the product is increased, and the intestinal system is regulated, making positive contributions to health.^21^ In recent studies, functional properties of products have been improved with the addition of natural ingredients with antimicrobial^22^ and antioxidant properties,^23^ apart from dietary fiber additives. Therefore, using cold-pressed byproducts is a good choice for producing functional muffins.

The purpose of this study was to ascertain the impact of incorporating OSB (5–20%) into the muffin recipe as a partial replacement for wheat flour on the physicochemical, textural, rheological, nutritional, and sensory characteristics of the muffin.

## 2 Materials and Methods

### 2.1 Materials

Wheat flour, sunflower oil, sugar, whole milk, eggs, and baking powder in muffin formulation were purchased from a local supermarket in İstanbul, Turkey. OSB was obtained from a local company (Oneva Food Co. Istanbul, Turkey). This byproduct has been taken from the same serial production because the chemical composition of cold-pressed oils is altered by variables such as product and manufacturing variances. The cold pressing machine utilized in the study is a screw press with dimensions of 710 mm in length and 260 mm in width, a motor power of 1.5 kW, an energy consumption of 400–850 W/h, and helical shaft gear transmission. It provides a wide range of seed processing characteristics, including over 200 seed processing capacities ranging from 1 to 50 kg per hour. The cold pressing machine has a capacity of 1080 kg of product per day.

The enzymes, namely, α-amylase, amyloglucosidase, pancreatin (from porcine pancreas, 8 × USP specifications), and invertase, used in in vitro glycemic index (GI) determination, and analytical grade chemicals were supplied by Sigma Chem. Co. (St. Louis, MO, USA). GOPOD reagent used for the determination of glucose was supplied by Megazyme International Ireland Ltd., Wicklow, Ireland). All reagents and solvents used in this study were ordered from Sigma-Aldrich, USA.

### 2.2 Methods

#### 2.2.1 Characterization of OSB and Wheat Flour

The moisture, oil, protein, and ash contents of wheat flour and OSB were determined with standard AOAC methods, numbered 934.01, 2003.05, 990.03, and 942.05, respectively. The total amounts of carbohydrate were calculated by subtracting the sum of moisture, oil, protein, and ash from 100%.

The total phenolic content (TPC) of the methanol extracts was identified according to the modified method described by Singleton and Rossi.^24^ The TPCs of OSB and white flour were measured at 760 nm by a spectrophotometer (Agilent 8453E UV–vis spectroscopy system) using Folin-Ciocalteu’s phenol reagent. TPC values were calculated as milligrams of gallic acid equivalents (GAE) per gram of samples. The DPPH· free radical scavenging of OSB and white flour was determined according to Shimada, Fujikawa, Yahara, and Nakamura.^25^ The antioxidant capacity of OSB and white flour was measured at 517 nm by a spectrophotometer (Agilent 8453E UV–vis spectroscopy system) using DPPH^•^ (2,2-diphenyl-1-picrylhydrazyl). The results of antioxidant capacities were expressed as a percent of inhibition of the DPPH^•^ radical (% IDPPH).

For determination of water-holding capacity (WHC) and oil-holding capacity (OHC) of OSB and wheat flour, 1 g of wheat flour and OSB was mixed with 10 mL of distilled water/sunflower oil for 1 min. The mixture was then centrifuged for 30 min at 10,000*g*. WHC and OHC were given as g water or oil trapped by g wheat flour and g OSB.^26^

#### 2.2.2 Preparation of Muffin Samples

The preparation of muffins was conducted according to same formulation of Gökşen and Ekiz (2021) but differently substituting wheat flour by 5–20% with OSB. For batter preparation, first, sugar (50 g) and eggs (60 g) were blended at a high speed for 2 min in a stand mixer (Kitchen Aid, USA). Then, liquid ingredients [oil (50 g) and milk (50 g)] were added to the batter. Following 2 min of mixing at a medium speed, the dry ingredients were placed in the mixer, and mixing was continued for 2 min at low speed. The batter was divided into 50 ± 0.5 g pieces, then placed into muffin cups, and baked in an electrical oven (FIMAK, Turkey) at 180 °C for 30 min. After baking, the muffins were allowed to cool at room temperature for 2 h before analysis.

#### 2.2.3 Rheological Characteristics of Muffin Batters

The flow behavior characteristics, dynamic viscosity, and three interval thixotropy test (3-ITT) rheological features of muffin batters were analyzed at 25.0 ± 0.1 °C by a temperature-controlled rheometer (MCR 302; Anton Paar, Sydney, NSW, Austria).

The flow behavior rheological properties of the muffin batters were measured by utilizing a parallel plate configuration having a gap of 0.5 mm with a 25 mm probe (PP25). The shear rate varied between 0.1 and 100 s^–1^. The sample was placed between the plates, and the test was performed. The shear stress versus shear rate were subjected to the power law model and nonlinear regression to characterize the flow behavior (eq 1).
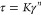
1

In eq 1, τ represents the shear stress (Pa), *K* represents the consistency index (Pa·s–n), γ represents the shear rate (1/s), and *n* represents the flow behavior index.

First, the linear viscoelastic region (LVR) was found employing an amplitude sweep test to assess dynamic oscillatory properties of muffin batters. The frequency sweep test between 0.1 and 10 Hz was conducted at a constant strain of 0.1% within LVR. The data set obtained from the test includes the storage modulus (*G*′) and loss modulus (*G*″). Power law model and nonlinear regression were subjected to evaluate parameters specific to rheological characteristics.

The samples were subjected to a dynamic rheological investigation using a parallel plate configuration. The LVR was first evaluated by using an amplitude sweep test with a strain value of 0.1%. The frequency sweep test was carried out in LVR at 0.1–10 Hz and 0.1–64 ω angular velocity ranges. The storage modulus (*G*′) and loss modulus (*G*″) were determined in addition to the angular velocity and frequency. Nonlinear regression and the power law model were employed to evaluate parameters related to detailed rheological characteristics.^27^
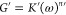
2
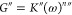
3

In eqs 2 and 3, *G*′(Pa), *G*′’ (Pa), ω (1/s), *K*′ and *K*″, and *n* correspond, respectively, to the storage modulus, loss modulus, angular velocity, consistency index values, and flow behavior index.

3-ITT test was applied to investigate structural regeneration of the muffin batters. In the first interval, muffin batters were analyzed at a low shear rate of 0.5 s^–1^ for 100 s. The muffin batters were deformed with a shear rate of 150 s^–1^ for 40 s in the second interval. In the third interval, the analysis was performed at the same conditions as the first interval at low shear rate.^28^

#### 2.2.4 Physicochemical Characteristics of Muffin Samples

The moisture, oil, protein, and ash contents of muffins were determined with standard AOAC methods, numbered 934.01, 2003.05, 990.03, and 942.05, respectively. The total amount of carbohydrate was calculated by subtracting the sum of moisture, oil, protein, and ash from 100%. A Novasina Lab Master aw meter (Novasina AG, Switzerland) was used to analyze the water activity (aw) of muffins at 30 °C.

#### 2.2.5 Crumb Structure, Height, Specific Volume, and Bake Loss

Digital images of the crumb were analyzed using ImageJ2x version 1.54c software (NIH, USA, https://imagej.nih.gov/ij/). The analysis involved interpreting the images based on the contrast differences between the pores and the solid phases. After cropping the images, the images were converted to grayscale and binarized once the threshold was reached to obtain the pore area and total pore area within the crumb (in square millimeters). Porosity was then calculated as the percentage of pores in the entire measured area.^29^ The height of the muffins was measured using calipers, from the base of each muffin to its highest point.^30^ All measurements were conducted in triplicate. For the measurement of loaf volumes, the rapeseed displacement method^31^ was employed, and the specific volume was calculated as the ratio of volume to weight, expressed in mL/g. The determination of weight loss involved weighing the initial cake dough and subsequently weighing the cake 1 h after baking.

#### 2.2.6 Textural Properties of Muffin Crumbs

Texture profile analysis (TPA) was conducted using a TA-TX plus Texture Analyzer (Stable Micro Systems, Surrey, UK), following a modified method by ref (30). 20 mm vertical slices of muffins were cut from the central crumb. The TPA parameters included a pretest speed of 1 mm/s, a test speed of 2.0 mm/s, and a post-test speed of 1.0 mm/s, with compression to 40% of the slice height and a 5 s interval between the two compression cycles. A trigger force of 5 g was applied, and a 25 mm cylindrical probe was used for the analysis. The parameters obtained from the TPA curves included hardness, chewiness, cohesiveness, and springiness. The samples were observed at 2 h after baking, as well as on the third and seventh days of storage.

#### 2.2.7 Fluorescence Measurements of Muffin Samples

In Maillard reaction (MR) studies, the fluorescence measurement is employed to quantify the formation of fluorescent advanced glycation end (AGE) products. The fluorescence of advanced Maillard products (FAMP) and the soluble tryptophan index (FAST index) are used to efficiently assess the extent of MR. Consequently, the FAST index is utilized to evaluate the MR products (MRP) in bread samples. Extraction of fluorescent substances was performed following the published procedure^32^ with slight modification. Specifically, 700 mg of homogenized bread samples was dissolved in 25 mL of 0.1 M borate buffer at pH 8.2. The soluble proteins were then separated by filtration through cellulose filter paper and used for the fluorescence measurement. A fluorescence spectrophotometer (Photon Technology International, NJ, USA) was utilized with excitation and emission wavelengths set at 290 and 340 nm for tryptophan fluorescence (F_TRP_) and at 320 and 395 nm for F_AMP_.

The FAST index was calculated as follows
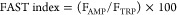


#### 2.2.8 In Vitro GI of Muffin Samples

The procedure of Demirkesen-Bicak, Arici, Yaman, Karasu, and Sagdic^33^ was used in order to determine the estimated glycemic index (eGI) of the muffin samples. 5 mL of deionized water was mixed with 1 g of homogenized muffins. The sample was then mixed with 10 mL of a pepsin-guar gum solution and incubated at 37 °C for 30 min in a water bath. 0.5 M sodium acetate solution (5.0 mL) was added following the incubation, and the pH was then adjusted to a range between 5 and 5.25. Pancreatin and amyloglucosidase (13.4 U/mL) enzyme solution was added, and the volume was then completed to 50 mL using deionized water. The sample was then incubated for 180 min in a water bath that was shaking. At 20, 30, 60, 90, 120, and 180 min of incubation, 0.5 mL of samples was collected and put in different test tubes. To allow the denaturation of the enzymes, the test tubes were heated in boiling water for 5 min. Deionized water was then used to increase the final amount to 5 mL, and for 5 min, the samples were centrifuged at 4000 g. Then, using a spectrophotometer (Shimadzu UV-1800, Japan) at a wavelength of 510 nm, the supernatant glucose content was determined using an assay kit GOPOD-format K-GLUC (Megazyme International Ireland Ltd.). Each sample’s hydrolysis index (HI) value was used to determine the eGI. The HI value was calculated by dividing the white bread area purchased from the neighborhood market’s area under the hydrolysis curve. According to Goñi, Garcia-Alonso and Saura-Calixto,^34^ the linear correlation was found between the HI and the GI. In the study, the potential relationship between responses to the same food in vitro and in vivo was investigated, and the following formula was used to determine the eGI
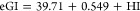


#### 2.2.9 Oxidative Stability

Using the OXITEST Device (Velp Scientifica, Usmate, MB, Italy), the oxidative stability of the muffin samples was evaluated in accordance with the procedure outlined in ref (35). The OXITEST device was utilized to treat the samples to an accelerated oxidation test. A 20 g sample was placed in the device’s receptacle, and the rapid oxidation test was conducted at 6 bar pressure and 90 °C. The induction period (IP) number (IP, in hours and minutes) was used as a measure of the sample’s redox stability.

#### 2.2.10 Sensory Analysis

Quantitative descriptive analysis was used to determine the sensory properties of muffin samples.^15^ A sensory panel was used for the quantitative descriptive analysis. The definitions for each descriptor are displayed in Table S2. The following descriptors were used: odor (typical and aromatic), flavor (typical, aromatic, and after-taste), mouth feel (moistness, oiliness, and chewiness), texture (hardness and springiness), and appearance (crumb porosity, crust darkness, and color). The sensory evaluation was carried out by 10 qualified panelists at Yildiz Technological University’s Food Engineering Department. Using 10 cm unstructured line scales, the descriptive scoring was assessed, and using a ruler, the scores were translated into numbers on a 10-point scale.

## 3 Results and Discussion

### 3.1 Physicochemical Characteristics of OSB and White Flour

The carbohydrate, protein, oil, moisture, and ash contents of OSB were 44.96, 32.34, 10.21, 7.51, and 4.98%, respectively (Table 1), indicating that OSB was rich in protein and carbohydrate content. In the study, different genotypes of okra seeds indicated various chemical composition within the range of 7.1–11.5% for moisture, 24.11–28.89% for oil, 37.4–40.7% for protein, 4.83–5.70% for ash, and 25.3–31.3% for carbohydrate.^36^ The reduced oil quantity in the OSB could be attributed to solvent extraction after cold pressing. On the other hand, the carbohydrate, protein, oil, moisture, and ash contents of white flour were determined as 73.47, 11.43, 1.50, 13.07, and 0.53%, respectively (Table 1).

**Table 1:** Physicochemical Characteristics of OSB and White Flour^a^

characteristics	OSB	white flour
carbohydrate (%)	44.96 ± 0.44	73.47 ± 0.09
protein (%)	32.34 ± 0.32	11.43 ± 0.06
oil (%)	10.21 ± 0.06	1.50 ± 0.05
moisture (%)	7.51 ± 0.02	13.07 ± 0.08
ash (%)	4.98 ± 0.02	0.53 ± 0.02
TPC (mg GAE/100 g)	128.03 ± 0.89	45.17 ± 0.07
IDPPH (%)	32.83 ± 0.35	8.39 ± 0.12
WHC (g water/g)	1.33 ± 0.03	0.67 ± 0.03
OHC (g oil/g)	0.65 ± 0.05	0.76 ± 0.04

a TPC: total phenolic compounds; IDPPH: inhibition percentage of the DPPH· radicals; WHC: water-holding capacity; OHC: oil-holding capacity; OSB: okra seed oil byproduct.

The TPC and IDPPH (%) values of OSB were found to be 128.03 mg GAE/100 g and 32.83%, respectively, while the TPC and IDPPH (%) values of white flour were 45.17 mg GAE/100 g and 8.39%, respectively. Petropoulos, Fernandes, Barros, Ciric, Sokovic, and Ferreira^36^ reported that the TPC contents of okra seeds ranged from 13.0 to 16.5 mg GAE/g extract, changing due to genotype and growing condition differences. The TPC content of wheat white flour was reported as 515 mg/kg (ferulic acid equivalent) by Li and Beta.^37^

WHC and OHC values of OSB were determined as 1.33 g water/g OSB and 0.65 g oil/g OSB, whereas WHC and OHC values of white flour were 0.67 g water/g white flour and 0.76 g oil/g white flour.

### 3.2 Rheological Properties of Muffin Batter

**Figure 1:**
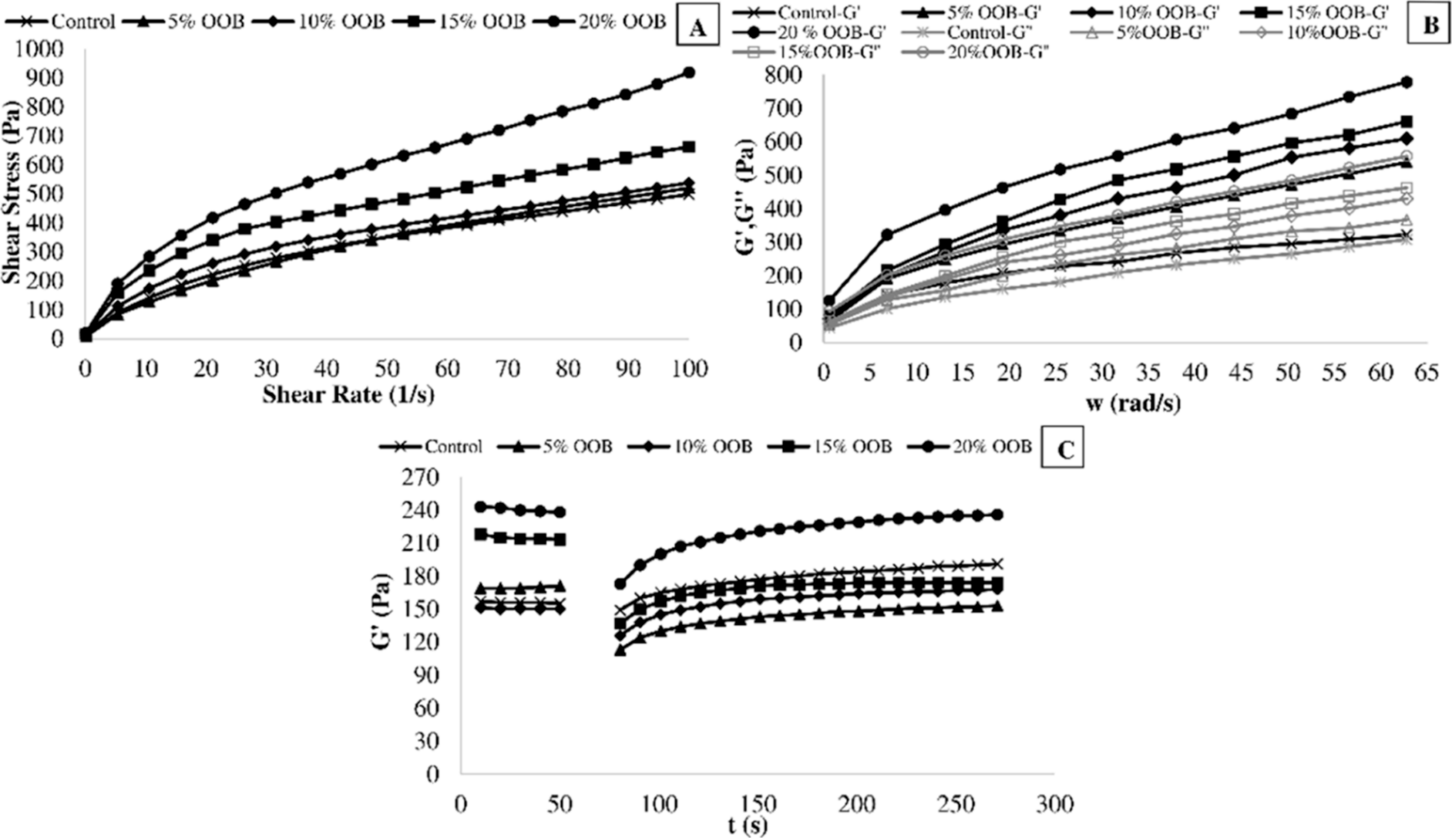
Rheological properties of cake batters. A: steady shear. B: dynamic. C: 3-ITT rheological properties [codes: control formulation (control) and formulation fortified with 5, 10, 15, and 20% cold-pressed OSB5 (OSB5, OSB10, OSB15, and OSB20, respectively)].

An ideal cake batter must be viscous enough to keep the additional air bubbles from ascending to the top and being lost during the initial heating.^38,39^ Also, the consistency of the cake batter has a substantial influence on the final cake’s quality. Shear stress values versus shear rate for control muffin batter and muffin batters prepared with different OSB concentrations are indicated in Figure 1A. The increase in the OSB content produced a progressive increase in the shear stress values. Figure 1A revealed that all samples showed a shear thinning behavior, indicating that the viscosity of all samples decreased with increasing shear rate. Table 2 indicates the values of the parameters (*K* and *n*) derived from the power law equation (*R*^2^ > 0.98). The *K* values of cake batters enriched with OSB ranged from 32.761 to 84.410 Pa s^*n*^, while the *K* value of control cake batter was 43.817 Pa s^*n*^. The increase in OSB content caused a significant increase in consistency values (*K*), explaining by the synergistic impact of the cake mix’s components. The *n* values of cake batters were between 0.432 and 0.602, and the *n* values were decreased as the content of OSB increased. The *n* values of cake batters were less than 1, indicating a non-Newtonian shear thinning behavior.

**Table 2:** Power Law Parameters Defining Steady Shear and Dynamic Rheological Properties of Muffin Batters^a^

	*K*	*n*	*R*^2^	*K*′	*n*′	*R*^2^	*K*″	*n*″	*R*^2^
control	43.82	0.528	0.999	66.45	0.381	0.999	36.62	0.508	0.997
OSB5	32.76	0.602	0.999	70.48	0.486	0.999	48.01	0.490	0.997
OSB10	58.98	0.479	0.999	74.46	0.507	0.999	51.88	0.506	0.998
OSB15	83.55	0.446	0.998	84.99	0.495	0.999	57.11	0.505	0.998
OSB20	84.41	0.432	0.999	139.14	0.408	0.998	80.42	0.459	0.996

a Codes: control formulation (control) and formulation fortified with 5, 10, 15, and 20% cold-pressed OSB (OSB5, OSB10, OSB15, and OSB20, respectively).

A frequency sweep test was utilized for determining the viscoelastic behavior of cake batters. The frequency dependence of the elastic (*G*′) and viscous (*G*″) moduli are shown in Figure 1B. In all samples, the *G*′ values were higher than the *G*″ values, revealing the behavior of a soft gel. This behavior is typical for cake batters.^40^ The control batter showed the lowest *G*′ and *G*″ values, and these parameters increased as the OSB content increased. In agreement with the flow behavior results, viscoelasticity reflects the existence of higher structural complexity in the OSB-containing batters than in the control batter. The *G*′ and *G*″ values obtained in response to angular velocity were modeled with the power law model, and the *K*′, *K*″, *n*′, and *n*″ values were obtained and are presented in Table 2. The *K*′ values (between 66.45 and 139.14) were higher than the *K*″ values (between 36.62 and 80.42) for all samples, and this result was another indication of the viscoelastic solid characteristic of the samples.

According to Figure 1C, all samples exhibit thixotropic behavior in the third period, indicating that all samples can regain their viscoelastic characteristic following high abrupt deformation during food processing. In the third interval, all samples exhibited thixotropic behavior, as shown in Figure 1C. These findings suggested that all samples might retain their viscoelastic characteristic during food preparation, which involves a large amount of sudden deformation.

### 3.3 Physicochemical Characteristics of Muffin Samples

The proximate composition (g/100 g on a wet basis) and aw of muffins are displayed in Table 3. The increasing trend was observed in fat, protein, and ash amount, while carbohydrate amount decreased with the addition of OSB. It was probably due to higher quantities of fat, protein, and ash in OSB. The moisture content of control sample is higher, which can be attributed to higher amount of moisture in white flour and lower bake loss (Table 3) in control bread. Similarly, the addition of coconut oilcake to muffin formulation increased protein, fat, and ash amount, whereas decreasing the carbohydrate and moisture amount which was also attributed to higher amount of fat and protein in oilcake.^41^ Grasso, Liu, and Methven^42^ also found that defatted sunflower seed flour addition (DSSF) led to an increase in the amounts of protein, fat, and ash and a decrease in carbohydrate and moisture values. In addition, water activities of DSSF-enriched muffins were lower compared to those of the control muffin. Similarly, control bread had the highest Aw value on all days, demonstrating that OSB enrichment decreased the amount of free water in the muffins. Soy and almond flour and whey protein enrichment of muffins decreased aw.^43^ Similarly, water activities of OSB fortified muffins have not differed from each other on first day, while OSB15 and OSB20 had the lowest aw on third and seventh days.

**Table 3:** Proximate Composition (g/100 g Wet Basis) and aw of Muffins^a^

						aw		
sample	moisture	fat	protein	ash	carbohydrate	first day	third day	seventh day
control	26.22 ± 0.03a	21.90 ± 0.06e	7.78 ± 0.01e	0.74 ± 0.03e	43.37 ± 0.07a	0.861 ± 0.001a	0.854 ± 0.001a	0.830 ± 0.001a
OSB5	25.45 ± 0.03c	22.06 ± 0.06d	8.26 ± 0.03d	0.93 ± 0.01d	43.29 ± 0.09a	0.855 ± 0.001b	0.844 ± 0.001b	0.824 ± 0.001bc
OSB10	25.48 ± 0.02c	22.38 ± 0.04c	8.66 ± 0.02c	1.04 ± 0.02c	42.43 ± 0.05b	0.855 ± 0.002b	0.844 ± 0.001b	0.825 ± 0.001b
OSB15	25.65 ± 0.06b	22.64 ± 0.04b	8.93 ± 0.04b	1.12 ± 0.03b	41.66 ± 0.13c	0.856 ± 0.002b	0.840 ± 0.001c	0.821 ± 0.001d
OSB20	25.72 ± 0.03^b^	23.30 ± 0.03^a^	9.22 ± 0.02^a^	1.21 ± 0.02^a^	40.55 ± 0.07^d^	0.856 ± 0.001^b^	0.840 ± 0.001^c^	0.822 ± 0.002^cd^

a Codes: control formulation (control) and formulation fortified with 5, 10, 15, and 20% cold-pressed OSB (OSB5, OSB10, OSB15, and OSB20, respectively).

### 3.4 Physical Characteristics of Muffin Samples

**Figure 2:**
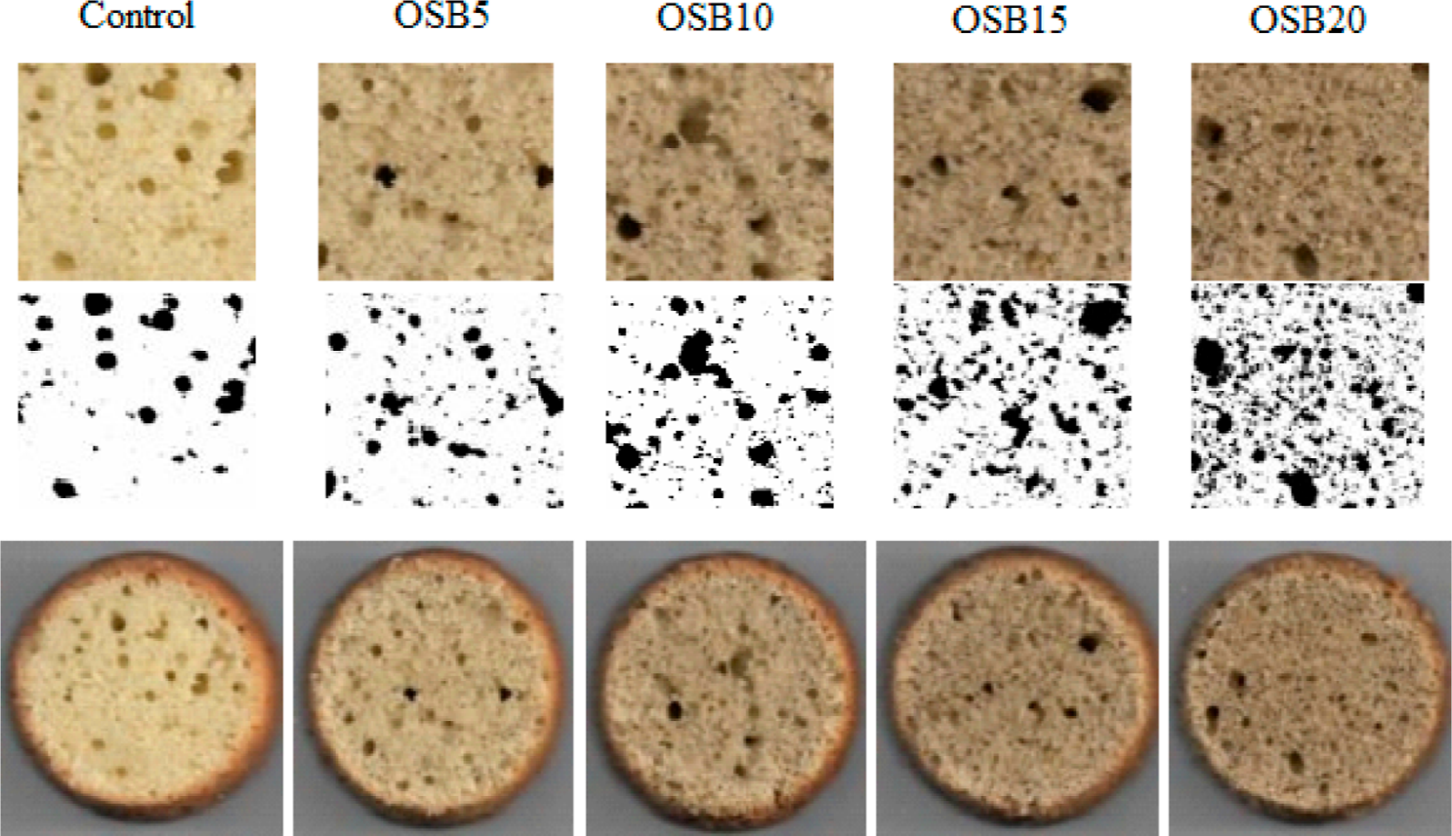
Crumb appearance and binarized image of muffins [codes: control formulation (control) and formulation fortified with 5, 10, 15, and 20% cold-pressed OSB5 (OSB5, OSB10, OSB15, and OSB20, respectively)].

Table 4 shows the physical characteristics of muffins. The addition of OSB did not influence specific volume and height of muffins significantly (*p* < 0.05). Bake loss of control bread was lowest in control bread, whereas bake loss of OSB-enriched muffins did not differentiate from each other. The addition of fruit byproducts also caused the higher bake loss.^44^ WHC increases with the addition of OSB and fiber-rich compounds, which causes more water to be lost during baking. Digital crumb appearance and binarized image of muffins are displayed in Figure 2, and from digital image analysis, porosity and circularity of muffins were calculated.

**Table 4:** Physical Characteristics of Muffins Fortified with OSB^a^

sample	specific volume (cm^3^)	height (cm)	bake loss (%)	porosity (%)	circularity	TPC (mg GAE/100 g)	IDPPH (%)
control	2.26 ± 0.08a	4.77 ± 0.06a	11.52 ± 0.36b	9.31 ± 0.05d	0.79 ± 0.01c	43.39 ± 0.74e	2.76 ± 0.22d
OSB5	2.33 ± 0.07a	4.83 ± 0.12a	12.59 ± 0.22a	10.24 ± 0.03d	0.84 ± 0.01a	49.29 ± 0.41d	3.55 ± 0.50cd
OSB10	2.32 ± 0.04a	4.73 ± 0.06a	12.38 ± 0.04a	14.77 ± 0.48c	0.84 ± 0.01a	52.58 ± 0.34c	4.77 ± 0.19bc
OSB15	2.30 ± 0.03a	4.73 ± 0.06a	12.55 ± 0.54a	19.27 ± 0.55b	0.80 ± 0.01bc	57.49 ± 0.49b	5.86 ± 0.35b
OSB20	2.25 ± 0.03^a^	4.67 ± 0.06^a^	12.19 ± 0.18^a^	25.38 ± 0.90^a^	0.82 ± 0.01^ab^	64.19 ± 0.83^a^	7.32 ± 0.21^a^

a Codes: control formulation (control) and formulation fortified with 5, 10, 15, and 20% cold-pressed OSB (OSB5, OSB10, OSB15, and OSB20, respectively).

The porosity of muffin crumbs increased with increasing rate of OSB. As a result of chia oil byproduct enrichment^29^ and aniseed oil byproduct addition,^15^ the porosity of muffins increased. It was attributed to increased amount of ash, fiber, and protein content. With the addition of OSB, the number of holes increased, increasing the porosity, which is consistent with the observed decrease in hardness. The circularity of a particle is a measurement of its resemblance to a perfect circle, which is close to 1 for perfect circles and close to 0 for irregular objects. The circularity of muffins ranged from 0.79 to 0.84, and the circularity of control muffin crumb was lower compared to muffins with OSB. The circularity values of muffins were found to be 0.76–0.77, which is close to our values; however, no significant difference was observed among control and chia seed oil byproduct-added muffins.^29^ As the OSB amount increased, TPC and IDPPH values also increased. Aranibar, Aguirre, and Borneo^29^ also showed that as the amount of added chia seed oil byproduct increased, TPC and total radical scavenging activity of muffins increased. In addition, upcycled sunflower flour enrichment led to an increase in antioxidant activity and TPC depending on the quantity of the flour.^45^ These were related to the higher amount of TPC and the antioxidant activity of added flours.

### 3.5 Texture Profile Analysis

The TPA of muffins fortified with varying amounts of OSB is displayed in Table 5 on the first, third, and seventh days of storage. The hardness of muffins ranged between 9.42 and 11.92 N on the first day. The results of TPA indicated a negative correlation between hardness and increasing addition of OSB. A similar trend was observed on the seventh day. Hardness values increased during storage, and the highest hardness value was observed in the control sample. In addition, the difference between the hardness values of the muffins during storage increased, indicating that the addition of OSB also slowed the hardening rate of muffins. This might be due to the high number of hydroxyl groups in the fiber structure, which resulted in additional water interaction through hydrogen bonding.^46^ Thus, increasing the OSB content of muffins could help to maintain their textural properties and extend their shelf life. Chewiness of fortified muffins also decreased proportionally to OSB amount and increased during storage according to arising amount of OSB. The changes in hardness and chewiness may be attributed to the dilution of gluten forming proteins. Similar effects of addition of fiber-rich components leading to gluten dilution on hardness and chewiness were reported.^47,48^ Although there was no significant difference among all muffins in terms of springiness and cohesiveness on 3 days analyzed (*p* < 0.05), both values decreased during the storage. Marchetti, Califano, and Andres^30^ also found that the fortification of muffin with pecan nut expeller meal had no significant effect on springiness up to 20%. Similarly, the addition of sesame oilseed cake up to 20% has not altered cohesiveness and springiness of muffins.^49^ Resilience values of muffins decreased during storage and differentiated from each other; however, no correlation was observed with the addition of OSB.

**Table 5:** TPA of Muffins Fortified with Okra Seed Byproduct^a^

parameters	storage day	control	OSB5	OSB10	OSB15	OSB20
hardness (N)	1	11.92 ± 0.24^a^	11.78 ± 0.24^a^	10.34 ± 0.06^b^	10.21 ± 0.04^b^	9.42 ± 0.14^c^
	3	22.82 ± 0.21^a^	20.53 ± 0.12^b^	19.37 ± 0.68^c^	18.23 ± 0.37^d^	17.86 ± 0.28^d^
	7	35.63 ± 1.06^a^	32.80 ± 0.21^b^	31.21 ± 0.14^c^	28.32 ± 0.59^d^	25.62 ± 0.17^e^
springiness	1	0.98 ± 0.02^a^	0,94 ± 0.02^a^	0.93 ± 0.03^a^	0.95 ± 0.01^a^	0.95 ± 0.01^a^
	3	0.93 ± 0.02^a^	0.94 ± 0.01^a^	0.93 ± 0.02^a^	0.92 ± 0.01^a^	0.91 ± 0.02^a^
	7	0.90 ± 0.02^a^	0.92 ± 0.01^a^	0.86 ± 0.01^a^	0.90 ± 0.03^a^	0.90 ± 0.01^a^
cohesiveness	1	0.77 ± 0.02^a^	0.76 ± 0.01^a^	0.78 ± 0.02^a^	0.78 ± 0.01^a^	0.79 ± 0.02^a^
	3	0.69 ± 0.01^a^	0.67 ± 0.02^a^	0.68 ± 0.01^a^	0.67 ± 0.02^a^	0.67 ± 0.02^a^
	7	0.62 ± 0.01^a^	0.62 ± 0.01^a^	0.60 ± 0.02^a^	0.61 ± 0.01^a^	0.64 ± 0.02^a^
chewiness	1	8.99 ± 0.06^a^	8.22 ± 0.36^b^	7.52 ± 0.11^c^	7.47 ± 0.04^c^	6.99 ± 0.08^d^
	3	14.46 ± 0.17^a^	12.62 ± 0.11^b^	11.88 ± 0.15^c^	11.52 ± 0.10^c^	12.36 ± 0.26^b^
	7	19.66 ± 0.60^a^	19.03 ± 0.09^a^	16.35 ± 0.18^b^	15.50 ± 0.23^b^	14.26 ± 0.27^c^
resilience	1	0.44 ± 0.01^a^	0.41 ± 0.01^b^	0.45 ± 0.01^a^	0.44 ± 0.01^a^	0.46 ± 0.01^a^
	3	0.36 ± 0.01^ab^	0.34 ± 0.01^b^	0.36 ± 0.01^ab^	0.37 ± 0.01^a^	0.35 ± 0.01^ab^
	7	0.30 ± 0.01^c^	0,33 ± 0.01^a^	0.31 ± 0.01^bc^	0.32 ± 0.01^ab^	0.33 ± 0.01^a^

a Codes: control formulation (control) and formulation fortified with 5, 10, 15, and 20% cold-pressed OSB (OSB5, OSB10, OSB15, and OSB20, respectively).

### 3.6 Fluorescence Measurements of Muffin Samples

Fluorescent MRPs have been interpreted in numerous model studies and food products in order to assess the rate and amount of MR.^50−52^ The FAST index is a quick, accurate, and inexpensive technique. The FAMP, such as pyrrole and imidazole derivatives, is used in this method to determine the level of MR.^53^ In our study, it was found that the FAST index decreased with increasing amount of OSB, indicating that OSB addition led to a decrease in the amount of MRP (Table 6). Similarly, enrichment of cakes with various spices decreased the FAST index, attributing to higher antioxidant ability of enriched cakes.^54^ In addition, the reducing sugar, TPC amounts, and antioxidant capacity of canihua were improved thanks to germination; thus, MRPs increased subsequently.^55^ In our study, antioxidative potential, TPC, and total carbohydrate also increased with increasing OSB, which causes a higher FAST index. The type and concentration of free amino acids and sugars, the temperature, the pH and the presence of a buffer, and the aw all affect the intensity of MR.^56^ An independent risk factor for chronic oxidative stress and inflammatory factor spikes in adulthood is associated with excessive MRP consumption.^57^ OSB addition has potential to prevent these MRP-related disorders.

**Table 6:** TRP Fluorescence, FIC Fluorescence, FAST Index Values, and eGI^a^

samples	TRP fluorescence (290/340 nm)	FIC fluorescence (340/420 nm)	FAST index (%)	eGI (%)
control	1141.77 ± 7.61^e^	1246.80 ± 15.32^d^	109.21 ± 2.07^a^	95.19 ± 0.30^a^
OSB5	1261.98 ± 29.12^d^	1298.48 ± 12.62^c^	102.94 ± 3.33^b^	94.14 ± 0.14^a^
OSB10	1598.56 ± 7.27^c^	1565.22 ± 22.84^b^	97.92 ± 1.57^b^	93.04 ± 0.80^a^
OSB15	1983.68 ± 8.11^b^	1690.25 ± 10.01^a^	85.21 ± 0.38^c^	90.50 ± 1.06^b^
OSB20	2052.03 ± 12.08^a^	1786.88 ± 14.92^b^	77.33 ± 0.62^d^	87.85 ± 0.07^c^

a TRP: tryptophan, FIC: fluorescent intermediary compounds, FAST index: fluorescence of advanced MRPs and soluble tryptophan. Codes: control formulation (control), formulation fortified with 5, 10, 15, and 20% cold-pressed OSB (OSB5, OSB10, OSB15, and OSB20, respectively). *TRP fluorescence is a measure of fluorescence intensity at λ_extinction_ = 290 nm and λ_emission_ = 340 nm. *FIC fluorescence is a measure FI at λ_extinction_ = 340 nm and λ_emission_ = 420 nm. FAST index is the ratio of FI of TRP and fluorescence multiplied by 100.

### 3.7 In Vitro GI of Muffin Samples

According to their possible impact on postprandial blood glucose levels (the “glycemic response”), foods containing carbohydrates can be categorized using the GI. In our study, even though eGI showed decreasing trend with increasing OSB addition, the fortified muffins more than 10% OSB had a reduced eGI significantly in comparison with control muffins (Table 6; *p* < 0.05). Nonstarchy substances in food products have an ability to influence eGI via creating organized starch structures or decreasing enzyme activity. For instance, the addition of insect flour reduced eGI of muffin samples from 92.22 to 80.74.^58^ This was attributed to an increased rate of protein; consequently, the interaction between protein and starch molecules decreased starch digestibility. Protein is able to form a continuous coating around the starch granules, which can impact the functional characteristics of starch such as the rate of gelatinization and digestion. In our study, the addition of OSB increased the protein amounts of the muffins. Okra seeds contain elevated level of fibers, and okra gums were used as soluble fiber source.^59^ Soluble fibers stimulate the formation of viscous protein-fiber-starch network which traps starch granules and restricts the release of glucose.^60^ Moreover, the increase in phenolic compounds may lead to inhibitory effect on α-amylase and α-glucosidase enzymes activities, which decrease eGI.^61^ Therefore, the decrease in eGI of OSB fortified muffins may be associated with increased levels of protein, fiber, and TPC.

### 3.8 Oxidative Stability

**Figure 3:**
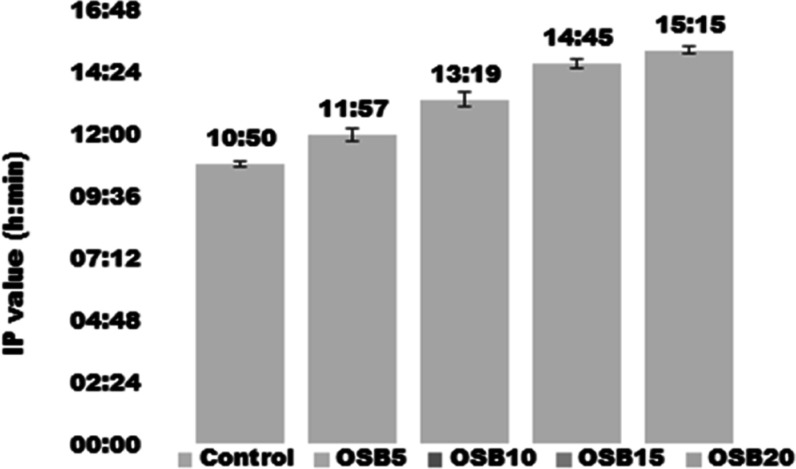
IP values of muffin samples.

Figure 3 shows the IP values of muffin samples at 90 °C and 6 bar. The IP values of the muffin samples containing OSB (between 11:57 and 15:15 h) were higher than the IP value of the control sample (10:50 h), indicating that OSB improved the oxidative stability of the muffin samples. The physicochemical analysis showed that OSB has considerable amount of TPC and strong antioxidant activity. The addition of OSB increased TPC value and antioxidant activity of the muffin samples. The increase in the IP value of samples can be explained by the interaction of the OSB phenolic with other antioxidant molecules.^62^

### 3.9 Sensory

**Figure 4:**
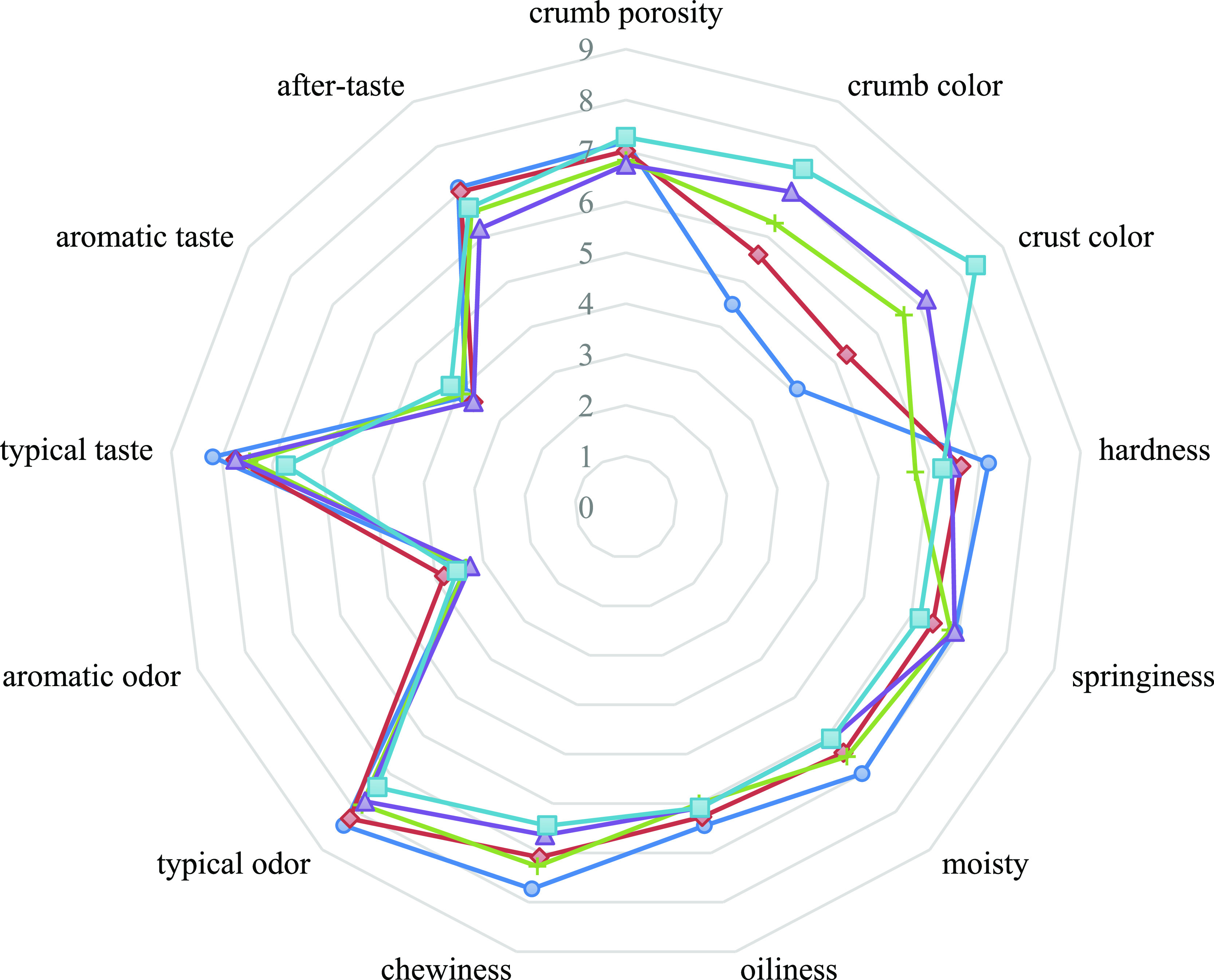
Sensory attributes of muffins (●, control; ⧫: OSB5; + : OSB10; ▲: OSB15; and ■: OSB20).

The sensory attributes of the muffins, which were determined as appearance, texture, mouth feel, odor, and taste, are presented in Figure 4 and Table 4. Muffins containing varying levels of OSB had significant effects on crumb color, crust darkness, hardness, chewiness, and typical odor. The darkness of muffin crumb and crust fortified with increasing OSB were noticed by the panelist, which is consistent with lightness values obtained from the colorimeter (Table S). Textural evaluation of panelists was in accordance with instrumental analysis data. The highest hardness and chewiness were perceived in control bread. The springiness of breads was not different from each other significantly (*p* < 0.05). Although muffins with increasing levels of OSB were perceived as less moist than the control, no significant differences were found. A typical odor of 20% OSB added bread was the lowest, indicating a high amount of OSB addition-altered typical baked product odor. However, no significant difference was observed in aromatic odors of muffins (*p* < 0.05). In terms of taste, control bread had the highest typical taste and after-taste values; in spite of that, no significant difference is present. It could demonstrate that control muffins provide more intense flavor. The addition of OSB has shown no unfavorable impact on sensory attributes considering texture, mouth fell, odor, and taste.

## 4 Conclusions

This study examined the effects of adding OSB to muffin batter at various concentrations, ranging from 0 to 20%. All muffin samples showed a shear thinning behavior, indicating that the viscosity of all samples decreased with increasing shear rate. All samples showed the viscoelastic solid characteristic. Muffins fortified with OSB indicated increased protein, oil, and ash contents. Porosity increased as the amount of OSB in muffin recipes increased, whereas height decreased. Also, it was found that the FAST index decreased with increasing amount of OSB, indicating that OSB addition led to a decrease in the amount of MRP. The fortified muffins with more than 10% OSB had a reduced eGI significantly in comparison with control muffins (*p* < 0.05), explaining that the decrease in eGI of OSB-fortified muffins may be associated with the increased levels of protein, fiber, and TPC. The IP values of the muffin samples containing OSB were higher than the IP value of the control sample, indicating that OSB improved the oxidative stability of the muffin samples. The addition of OSB has shown no unfavorable impact on sensory attributes considering texture, mouth fell, odor, and taste.

## Supporting Information Available

Color characteristics of muffins and descriptor definitions (PDF)
