# Supplementary material for: Cold-Pressed Okra Seed Oil Byproduct as an Ingredient for Muffins to Decrease Glycemic Index, Maillard Reaction, and Oxidation
Source: ACS Omega. 2024 Feb 9;9(7):7491–501. doi: 10.1021/acsomega.3c06027 (PMC10882597; doi:10.1021/acsomega.3c06027)
Supplement: Supplementary file 1 — ao3c06027_si_001.pdf [file ao3c06027_si_001.pdf]

Table S1 The descriptor definitions utilized in quantitative sensory analysis

**Descriptor**	**Definition**	**Scale measures**
**Appearance**		
Crumb porosity	Number and homogeneity of gas bubbles	Low (None)- High (Extreme)
Crust color	Level of crust darkness	Low (Light) - High (dark)
Crumb color	The degree of browning of the crumb	Low (Light) - High (dark)
**Texture**		
Hardness	Applying pressure with a finger to compress the muffin	Low (Soft) - High (Hard)
Springiness	The rate at which a crumb recovers once a finger is released	Low (Inelastic) - High (Elastic)
**Mouth feel**		
Moisty	detected wetness from the muffin in the mouth	Low (Dry) - High (Moist)
Oiliness	Sensed oiliness after eating the muffin	Low (Not perceived) - High (Intense)
Chewiness	difficulty completely chewing the muffin	Low (difficult to chew) - High (easy to chew)
**Odor**		
Typical	Typical baked-food aroma, mostly sweet and fresh	Low (foreign) - High (typical)
Aromatic	The strength of the product's aromatic odor	Low (Not perceived) – High (Intense)
**Taste**		
Typical	Typical baked food taste: fresh, sweet, and fatty	Low (Uncharacteristic) - High (Typical)
Aromatic	The taste is sweet and aromatic, slightly pungent	Low (Not perceived) - High (Intense)
After-taste	The strength of olfactory and/or gustatory sensation that follows swallowing	Low (Disagreeable) - High (Agreeable)

Table S2 Color characteristics of muffins based on *L**, *a**,*b** scale

Sample	*L**		*a**		*b**	
	**Crumb**	**Crust**	**Crumb**	**Crust**	**Crumb**	**Crust**
Control	74.72 ± 0.74^{a}	61.64 ± 0.34^{a}	-2.38 ± 0.16^{d}	9.61 ± 0.29^{a}	25.96 ± 0.36^{a}	35.68 ± 0.56^{a}
OSB5	64.80 ± 1.21^{b}	55.41 ± 0.41^{b}	0.03 ± 0.04^{c}	10.42 ± 0.79^{a}	20.09 ± 0.67^{b}	29.51 ± 1.02^{b}
OSB10	59.94 ± 0.42^{c}	52.18 ± 0.84^{c}	1.50 ± 0.18^{b}	10.06 ± 0.31^{a}	18.23 ± 0.17^{c}	27.06 ± 0.47^{c}
OSB15	57.11 ± 0.25^{d}	50.83 ± 0.14^{cd}	2.49 ± 0.20^{a}	10.51 ± 0.21^{a}	16.09 ± 0.39^{d}	23.64 ± 0.37^{d}
OSB20	52.32 ± 1.03^{e}	50.18 ± 0.61^{d}	2.65 ± 0.09^{a}	6.08 ± 0.05^{b}	13.24 ± 0.13^{e}	19.09 ± 0.24^{e}

Codes: Control formulation (Control), formulation fortified with 5, 10, 15, and 20% cold-pressed okra seed by-product (OSB5, OSB10, OSB15, OSB20, respectively).
